# The interaction between N-terminal pro-brain natriuretic peptide and fluid status in adverse clinical outcomes of late stages of chronic kidney disease

**DOI:** 10.1371/journal.pone.0202733

**Published:** 2018-08-22

**Authors:** Yi-Chun Tsai, Hui-Ju Tsai, Chee-Siong Lee, Yi-Wen Chiu, Hung-Tien Kuo, Su-Chu Lee, Tzu-Hui Chen, Mei-Chuan Kuo

**Affiliations:** 1 Division of General Medicine, Kaohsiung Medical University Hospital, Kaohsiung, Taiwan; 2 Division of Nephrology, Department of Internal Medicine, Kaohsiung Medical University Hospital, Kaohsiung, Taiwan; 3 Faculty of Renal Care, College of Medicine, Kaohsiung Medical University, Kaohsiung, Taiwan; 4 Department of Family Medicine, Kaohsiung Municipal Ta-Tung Hospital, Kaohsiung, Taiwan; 5 Division of Cardiology, Kaohsiung Medical University Hospital, Kaohsiung, Taiwan; 6 Department of Nursing, Kaohsiung Medical University Hospital, Kaohsiung, Taiwan; International University of Health and Welfare, School of Medicine, JAPAN

## Abstract

**Introduction:**

Fluid overload is one of the major characteristics and complications in patients with chronic kidney disease (CKD). N-terminal pro-brain natriuretic peptide (NT-proBNP) is related to fluid status and fluid distribution. The aim of this study is to investigate the interaction between NT-proBNP and fluid status in adverse clinical outcomes of late stages of CKD.

**Methods:**

We enrolled 239 patients with CKD stages 4–5 from January 2011 to December 2011 and followed up until June 2017. Fluid status was presented as hydration status (HS) value measured by body composition monitor, while HS>7% was defined as fluid overload. Clinical outcomes included renal outcomes (commencing dialysis and estimated glomerular filtration rate decline>3 ml/min/1.73 m^2^/year), all-cause mortality and major adverse cardiovascular events (MACEs).

**Results:**

During a mean follow-up of 3.3±2.0 years, 129(54.7%) patients commenced dialysis, 88(37.3%) patients presented rapid renal function decline, and 48(20.3%) had MACEs or died. All patients were stratified by HS of 7% and the median of plasma NT-proBNP. The adjusted risks for commencing dialysis was significantly higher in patients with high plasma NT-proBNP and HS>7% compared to those with low plasma NT-proBNP and HS≦7%. There was a significant interaction between plasma NT-proBNP and HS in commencing dialysis (P-interaction = 0.047). Besides, patients with high plasma NT-proBNP and HS>7% had greater risks for MACEs or all-cause mortality than others with either high plasma NT-proBNP or HS>7%.

**Conclusion:**

NT-proBNP and fluid overload might have a synergistic association of adverse clinical outcomes in patients with late stages of CKD.

## Introduction

Brain natriuretic peptide (BNP) is a cardiac neurohormone synthesized in the cardiac myocytes in response to increased left ventricular (LV) wall stress and stretch [1, 2]. Pre-proBNP is released from BNP and subsequently cleaved into proBNP, that is further cleaved to biologically active BNP and inactive N-terminal proBNP (NT-proBNP) [3, 4]. NT-proBNP has been considered as not only a biomarker of LV dysfunction [5] but also a predictor of major adverse cardiovascular events (MACEs) and all-cause mortality in dialysis patients as well [6, 7]. In chronic kidney disease (CKD) patients not on dialysis, accumulating evidence presents a significant association between plasma NT-proBNP level and all-cause mortality and MACEs [8–10]. Besides, elevated plasma NT-proBNP level indicates an increased risk for initial dialysis [11, 12]. NT-proBNP has been a potential biomarker of adverse clinical outcomes in CKD patients both on dialysis or not.

Fluid overload is one of the major characteristics and complications in CKD patients. Fluid overload is significantly associated with faster decline of renal function and increased risks for cardiovascular events or all-cause mortality [13, 14]. Plasma BNP is related to fluid status [15] and fluid distribution between the intracellular water (ICW) and extracellular water (ECW) [16]. The interaction between NT-proBNP and fluid status may be associated with adverse clinical outcomes. NT-proBNP and fluid status may have a synergistic effect on prognostic implications in CKD patients. Thus, the aim of this study is to assess whether the interaction between NT-proBNP and fluid status is associated with adverse clinical outcomes, including commencing dialysis, MACEs and all-cause mortality in patients with CKD stages 4–5 not on dialysis.

## Materials and methods

### Study participants

Two hundred and thirty-nine patients with CKD stages 4–5 were invited to participate in the study from January 2011 to December 2011 at one hospital in Southern Taiwan. CKD was staged according to Kidney Disease Outcomes Quality Initiative (K/DOQI) definition and the estimated glomerular filtration rate (eGFR) was calculated using the equation of the 4-variable Modification of Diet in Renal Disease (MDRD) Study (CKD stage 4, eGFR: 15–29 ml/min/1.73m^2^; CKD stage 5, eGFR<15 ml/min/1.73m^2^) [17]. The exclusion criteria of this study included patients with eGFR ≥30 ml/min/1.73m^2^ and patients having underline disease with malignancy. We also excluded three patients with less than three eGFR measurements during follow-up period because of minimal requirement of eGFR slope calculation. The study protocol was approved by the Institutional Review Board of the Kaohsiung Medical University Hospital (KMUH-IRB-990125). The methods were carried out in accordance with the relevant guidelines, including any relevant details, such as low risk for study design, private protection, and safe management. Informed consents were obtained in written form from patients and all clinical investigations were conducted according to the principles expressed in the Declaration of Helsinki.

### Quantification of circulating NT-proBNP

Blood samples were collected at enrollment. All blood samples were aliquoted and stored in a -80°C freezer for the analysis of NT-proBNP after finishing recruitment. Plasma NT-proBNP was measured in duplicate using commercial enzyme-linked immunosorbent assays (Elecsys*®* Roche) based on the instructions of the manufacturer. The reportable range of NT-proBNP assay was 5–35000 pg/mL Intraassay and interassay coefficients of variation of NT-proBNP were 1.03% and 2.56% respectively.

### Measurement of fluid status

Fluid status was measured once at enrollment by Body Composition Monitor (BCM, Fresenius Medical Care), a bioimpedance spectroscopy method as in our previous study [13, 14]. BCM provides the data of normohydrated lean tissue, normohydrated adipose tissue, and excess fluid mass in whole body based on the difference of impedance in each tissue through 3-component tissue-based model [18, 19]. Overhydration (OH) value, as an absolute fluid status, can be calculated from the difference between the normal expected and measured ECW [19]. The BCM can detect more precise body fluid compartment, and has been validated in the general population and CKD patients on dialysis [20–22]. The fluid status was also validated in CKD patients not on dialysis, and Hung et al. defined fluid overload as hydration status (HS, OH/ECW) value over 7% [20]. Therefore, we defined fluid overload as HS>7% in the present study.

### Data collection

Participants were asked to fast for at least 12 hours before blood sample collection for the biochemistry study and protein in urine was measured by urine protein-creatinine ratio (PCR). Demographic and clinical data were obtained from medical records and interviews with patients at enrollment. Information regarding patient medications including diuretics, β-blockers, calcium channel blockers, angiotensin converting enzyme inhibitors (ACEI), angiotensin II receptor blockers (ARB), and statin within 3 months before enrollment was obtained from medical records. Patients were classified as diabetic by history and blood glucose values using the American Diabetes Association criteria, oral hypoglycemia agent use, or insulin use. Cardiovascular disease was defined as a history of acute or chronic ischemic heart disease, myocardial infarction, and heart failure. Cerebrovascular disease was defined as a history of cerebral infarction or hemorrhage.

### Clinical outcomes

Participants were contacted at outpatient clinics at 3-month intervals to evaluate the clinical status. The renal outcomes consisted of commencing dialysis (hemodialysis or peritoneal dialysis) and rapid renal function decline. Commencing dialysis was confirmed by reviewing catastrophic illness certificate (issued by the Bureau of National Health Insurance in Taiwan) and medical charts. Rapid renal function decline was defined as the eGFR decline > 3 ml/min/1.73 m^2^ per year (eGFR slope). The eGFR slope was calculated by the regression coefficient between eGFR and time in units of ml/min per 1.73 m^2^ per year based on all eGFR values available from enrollment to the end of the observation period. At least three eGFR values were required to estimate the eGFR slope. Rapid eGFR decline was defined as the lowest quartile (the eGFR decline more than 3 ml/min/1.73m^2^ per year, an integer near the cutoff point between the lowest two quartiles of the eGFR slope) [13]. MACEs were defined as new onset of myocardial infarction, unstable angina, acute hemorrhagic or ischemic stroke, hospitalizations related to acute phase of congestive heart failure or arrhythmia. The information of death obtained by direct contact with patients and families was further supplemented by reviewing medical records and screening the data bank of the National Mortality File. We excluded MACEs or death after commencing dialysis. Patients were censored at last contact or the end of observation in June 2017.

### Statistical analysis

The study population was further classified into four groups according to HS value of 7% and the median of circulating NT-proBNP. Continuous variables were expressed as mean±SD or median (25^th^, 75^th^ percentile), as appropriate, and categorical variables were expressed as percentages. Skewed distribution continuous variables were log-transformed to attain normal distribution. The significance of differences in continuous variables between groups was tested using the one-way analysis of variance (ANOVA) followed by the post hoc test adjusted with a Bonferroni correction or the Kruskal-Wallis H test, as appropriate. The difference in the distribution of categorical variables was tested using the Chi-square test. The association between fluid status and NT-proBNP was examined by linear regression. Time-to-event survival analysis by Kaplan-Meier survival curve was used to test fluid status or circulating NT-proBNP level as a predictor of the risk of commencing dialysis and composite outcomes either MACEs or all-cause mortality. Cox regression models were utilized to evaluate the interaction between fluid status and circulating NT-proBNP level in commencing dialysis. Multivariable logistic regression models were also utilized to examine the association of rapid renal function decline with the interaction between fluid status and circulating NT-proBNP level. Age, gender, diabetes mellitus, cardiovascular disease, diuretics usage, ACEI/ARB usages, eGFR, and urine PCR were selected in multivariate analysis for renal outcomes. In addition to above parameters, statin usage and low-density lipoprotein were put in multivariate analysis for MACEs or all-cause mortality. P-value for interaction was utilized to analyze whether a synergistic effect between fluid status and circulating NT-proBNP on clinical outcomes existed in all patients. Statistical analyses were conducted using SPSS 18.0 for Windows (SPSS Inc., Chicago, Illinois). Statistical significance was set at a two-sided p-value of less than 0.05.

## Results

### Characteristics of entire cohort

The comparison of clinical characteristics between groups based on HS value at 7% and the median of circulating NT-proBNP level (261.8pg/ml) is shown in Table 1. Of all patients, the mean age was 64.9±11.8 years, 53.0% were male, and the mean of HS was 7.8%. Ninety-three (39.4%) and 39 (16.5%) had diabetes and cardiovascular disease respectively. The patients with HS>7% and high circulating NT-proBNP level had the highest proportion of cerebral vascular disease and treatment with calcium channel blocker, β-blocker and diuretics than other groups. Systolic blood pressure, blood urea nitrogen and urine PCR were higher, and diastolic blood pressure, eGFR, serum albumin, hemoglobin and calcium levels were lower in patients with both HS>7% and high circulating NT-proBNP level than other groups (Table 1).

**Table 1:** The clinical characteristics of study subjects stratified by plasma N-terminal pro-brain natriuretic peptide (NT-proBNP) and fluid status.

	Entire Cohort N = 236	HS≦7% NT-proBNP≦median N = 85	HS≦7% NT-proBNP>median N = 46	HS>7% NT-proBNP≦median N = 33	HS>7% NT-proBNP >median N = 72	P-value
Demographics						
Age (year)	64.9±11.8	64.5±10.6	64.0±13.4	64.0±13.2	66.4±11.4	0.617
Sex (male, %)	53.0	61.2	32.6	60.6	52.8	0.013
Smoke (%)	18.2	18.8	17.4	12.1	20.8	0.754
Alcohol (%)	10.2	14.1^&^	10.9	9.1	5.6*	0.363
Cardiovascular disease (%)	16.5	11.8	19.6	9.1	23.6	0.130
Cerebral vascular disease (%)	9.3	2.4^&^	8.7	15.2	15.3*	0.026
Hypertension (%)	81.4	80.0	76.1	84.8	84.7	0.626
Diabetes mellitus (%)	39.4	31.8	30.4	51.5	48.6	0.042
Body mass index (kg/m^2^)	24.4±3.8	24.5±3.7	24.8±4.0	24.7±3.4	24.0±3.8	0.660
Systolic blood pressure (mmHG)	138.3±19.1	132.5±16.1^&^	137.9±19.0	142.4±17.8	143.5±21.1*	0.002
Diastolic blood pressure (mmHG)	75.9±11.4	78.3±10.5^&^	75.6±12.0	76.7±11.8	73.1±11.4*	0.040
Mean blood pressure (mmHG)	96.7±11.4	96.3±10.5	96.3±12.2	98.6±11.1	96.7±12.1	0.802
CKD stage 4 (%)	52.1	67.1	32.6	63.6	41.7	<0.001
5 (%)	47.9	32.9	67.4	36.4	58.3	
Medications						
Calcium channel blocker (%)	58.1	47.1	45.7	66.7	75.0	0.001
β-blocker (%)	24.2	14.1	34.8	9.1	36.1	0.001
ACEI/ARB (%)	52.5	51.8	52.2	66.7	47.2	0.323
Diuretics (%)	22.0	11.8^&^	21.7	21.2	34.7*	0.007
Statin (%)	29.7	29.4	45.7^&^	36.4	16.7^†^	0.007
Body composition						
Lean tissue index (kg/m^2^)	13.8±2.7	14.7±2.7^†^^&^	13.3±2.6*	13.4±2.2	13.2±2.7*	0.001
Fat tissue index (kg/m^2^)	10.0±4.3	9.7±4.4	11.2±4.8	10.3±3.5	9.5±4.2	0.157
HS (OH/ECW, %)	7.8±8.6	1.8±4.1^#^^&^	1.9±3.7^#^^&^	13.5±5.0*^†^	16.2±7.2*^†^	<0.001
Laboratory parameters						
NT-proBNP (pg/ml)	261.8(124.5,742.1)	102.1(55.2,180.7)^†^^&^	486.8(321.0,989.7)^†^^&^	148.8(100.2,189.4)*^&^	1060.0(425.5,1753.8)*^†^^#^	<0.001
Blood urea nitrogen (mg/dl)	44.8(34.0,62.9)	37.0(30.4,52.6)^†^^&^	55.7(33.9,69.3)*	40.0(32.0,59.4)^&^	51.7(40.6,71.8)*^#^	<0.001
eGFR (ml/min/1.73m^2^)	15.8±7.7	18.3±7.6^†^^&^	13.8±8.0*	17.4±6.8	13.5±6.9*^#^	<0.001
Glycated hemoglobin (%)	5.8(5.5,6.6)	5.8(5.5,6.3)	5.7(5.5,6.6)	6.0(5.5,7.1)	5.9(5.5,6.8)	0.600
Hemoglobin (g/dl)	10.5±1.8	11.2±1.7^&^	10.3±1.7*	10.6±1.7	9.8±1.8	<0.001
Albumin (g/dl)	4.1±0.4	4.3±0.3^&^	4.1±0.3^&^	4.1±0.3^&^	3.9±0.5*^†^^#^	<0.001
Calcium (mg/dl)	9.0±0.6	9.2±0.4^&^	9.1±0.7^&^	9.3±05^&^	8.8±0.6*^†^^#^	<0.001
Phosphate (mg/dl)	4.2(3.8,5.0)	4.0(3.6,4.5)^†^^&^	4.3(3.9,5.5)*	4.3(3.9,4.8)	4.3(3.9,5.1)*	0.022
Uric acid (mg/dl)	7.6±1.6	7.4±1.5	7.9±2.1	7.9±1.4	7.6±1.5	0.257
Cholesterol (mg/dl)	176.5(152.8,210.3)	178.0(156.0,211.0)	186.5(161.5,216.3)	177.0(156.0,204.0)	168.0(140.8,212.3)	0.282
Triglyceride (mg/dl)	115.5(76.8,165.0)	115.0(74.0,176.0)	122.0(98.0,155.5)	141.0(85.5,175.5)	100.0 (71.3,146.5)	0.275
Low-density lipoprotein (mg/dl)	101.3±31.7	102.6±40.0	104.8±29.5	93.8±25.5	100.9±36.4	0.879
High-density lipoprotein (mg/dl)	44.2±14.4	43.6±14.1	45.5±13.9	43.0±12.8	44.4±15.9	0.504
hsCRP (mg/L)	1.4(0.7,3.8)	1.3(0.7,3.6)	1.8(1.1,4.6)	1.0(0.4,2.7)	1.5(0.6,4.0)	0.414
Urine protein/creatinine ratio	1.9±2.1	1.0±0.9	1.5±1.2	1.9±1.7	3.0±3.0	<0.001
Urine protein/creatinine ratio>1mg/mg (%)	56.9	40.5^†^^&^	58.1*	53.1^&^	76.1*^#^	<0.001

Data are expressed as number (percentage) for categorical variables and mean±SD or median (25^th^, 75^th^ percentile) for continuous variables, as appropriate.

Abbreviations: ACEI/ARB, angiotensin converting enzyme inhibitors/angiotensin II receptor blockers; HS, hydration status; eGFR, estimated glomerular filtration rate; hsCRP, high sensitivity c-reactive protein.

*P < 0.05 compared with HS≦7% and NT-proBNP≦median group

^†^*P* < 0.05 compared with HS≦7% and NT-proBNP >median group

^#^*P* < 0.05 compared with HS>7% and NT-proBNP≦median group

^&^*P* < 0.05 compared with HS>7% and NT-proBNP >median group

The median of NT-proBNP cut at 261.80 pg/ml

### The correlation between NT-proBNP and fluid status

**Fig 1:**
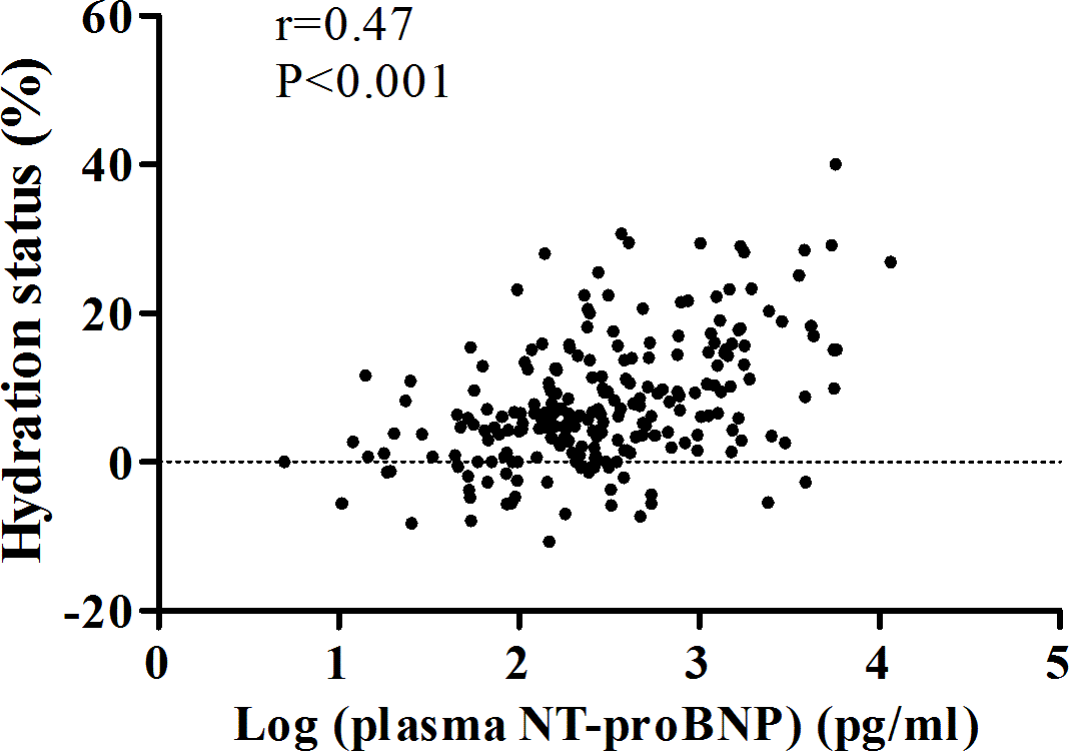
The correlation between plasma N-terminal pro-brain natriuretic peptide (NT-proBNP) and fluid status.

The positive correlation between circulating NT-proBNP and fluid status is shown in Fig 1. Elevated NT-proBNP level was significantly correlated with high HS (unstandardized β: 4.24, 95% Confidence Interval (CI): 2.44–6.05, P<0.001) in multivariate linear regression (Table 2). The highest tertile of circulating NT-proBNP level was significantly associated with HS as compared with the lowest tertile of circulating NT-proBNP level (unstandardized β: 3.96, 95%Cl: 1.25–6.66, P = 0.004) after age, sex, diabetes mellitus, cardiovascular disease, diuretics usage, eGFR, urine PCR, and serum albumin and hemoglobin levels. There was a dose dependent correlation between circulating NT-proBNP level and fluid status.

**Table 2:** The association of plasma N-terminal pro-brain natriuretic peptide (NT-proBNP) with fluid status.

Unstandardized β(95%Cl)	Unadjusted model	Model 1	Model 2	Model 3
Log(NT-proBNP)	6.76(5.12,8.40)^#^	7.10(5.47,8.72)^#^	6.66(4.98,8.33)^#^	4.24(2.44–6.05)^#^
NT-proBNP				
Terrtile 1	Reference	Reference	Reference	Reference
Terrtile 2	3.34(0.84,5.83)^&^	3.78(1.31,6.25)^&^	3.58(1.18,5.98)^&^	2.00(-0.37,4.36)
Terrtile 3	8.02(5.52,10.51)^#^	8.55(6.06,11.03)^#^	7.72(5.22,10.21)^#^	3.96(1.25–6.66)^&^

Unadjusted model is as no adjustment of other covariates

Multivariate model 1 is adjusted for age and sex

Multivariate model 2 comprises model 1 as well as diabetes mellitus, heart disease, and diuretics usage

Multivariate model 3 comprises model 2 as well as estimated glomerular filtration rate, urine protein-creatinine ratio, and serum albumin and hemoglobin levels.

NT-proBNP tertile cut at 158.63 and 443.84pg/ml

^&^P<0.01, and

^#^P<0.001 compared with reference

### Fluid status, NT-proBNP and renal outcomes

**Fig 2:**
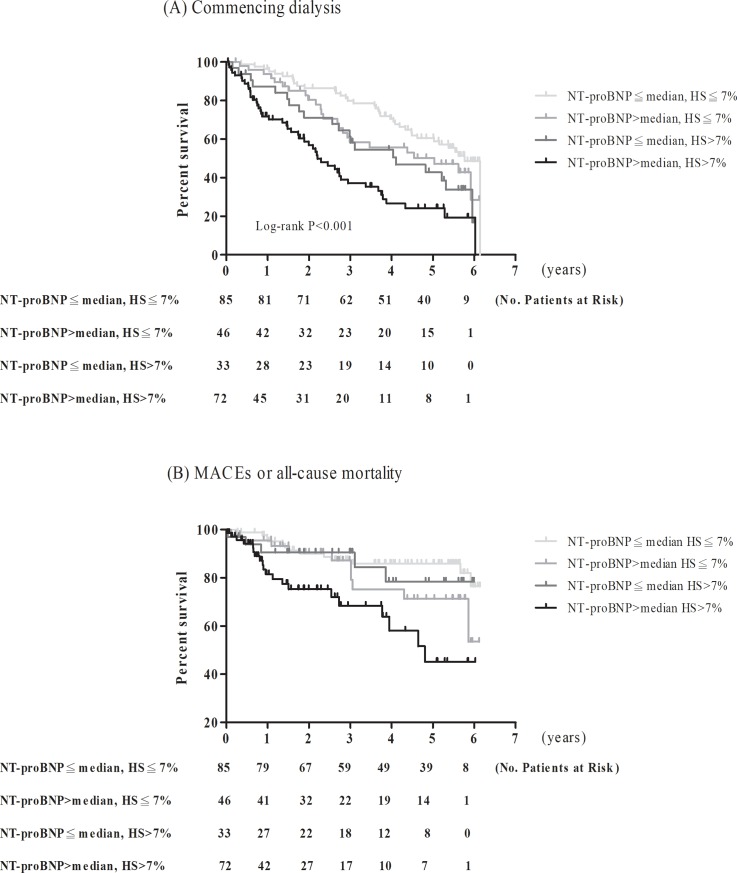
Kaplan-Meier survival curve for (A) commencing dialysis and (B) major adverse cardiovascular events or all-cause mortality in study subjects stratified by plasma N-terminal pro-brain natriuretic peptide (NT-proBNP) and fluid status.

One hundred and twenty-nine patients (54.7%) commenced dialysis during a mean follow-up of 3.3±2.0 years (Table 3). Patients with both HS>7% and high circulating NT-proBNP level had the highest proportion of commencing dialysis among 4 groups (P = 0.043, Table 3). Kaplan-Meier curves showed patients with both HS>7% and high circulating NT-proBNP level were more likely to commence dialysis compared to others (Fig 2A). We further analyzed the interaction between fluid status and circulating NT-proBNP level in commencing dialysis (Table 4). The unadjusted and adjusted HRs for commencing dialysis in patients with both HS>7% and high circulating NT-proBNP level compared with those with HS≦7% and low circulating NT-proBNP level was 3.32 (95% CI: 2.14–5.16) and 1.71 (95% CI: 1.03–2.85). There was a significant synergic association of fluid status and circulating NT-proBNP with commencing dialysis in patients with CKD stages 4–5 (HR: 2.09, 95%CI: 1.01–4.30, P-interaction = 0.047).

**Table 3:** Events of study subjects stratified by plasma N-terminal pro-brain natriuretic peptide (NT-proBNP) and fluid status.

	Entire Cohort N = 236	HS≦7% NT-proBNP≦median N = 85	HS≦7% NT-proBNP>median N = 46	HS>7% NT-proBNP≦median N = 33	HS>7% NT-proBNP >median N = 72	P-value
Follow-up time, year	3.3±2.0	4.2±1.7^&^	3.4±1.9^&^	3.4±1.9^&^	2.2±1.7*^†^^#^	<0.001
Dialysis, n(%)	129(54.7)	38(44.7)^&^	23(50.0)	21(63.6)	47(65.3)*	0.043
eGFR slope, ml/min/1.73m^2^	-2.3(-4.1,-1.1)	-1.8(-3.2,-0.9)	-1.6(-2.9,-0.9)	-2.6(-5.4,-1.0)	-3.1(-6.1,-1.4)	0.001
Rapid eGFR decline, n(%)^a^	88(37.3)	25(29.4)^&^	11(23.9)^#^^&^	16(48.5)^†^	36(50.0)*^†^	0.006
MACEs + all-cause mortality, n(%)	48(20.3)	13(15.3)	10(21.7)	5(15.2)	20(27.8)	0.222
MACEs, n(%)	31(13.1)	10(11.8)	5(10.9)	3(9.1)	13(18.1)	0.503
All-cause mortality, n(%)	23(9.7)	5(5.9)	5(10.9)	2(6.1)	11(15.3)	0.210

^a^Defined as eGFR decline >3 ml/min/1.73m^2^ per year

Abbreviations: HS, hydration status; eGFR, estimated glomerular filtration rate; MACE, major adverse cardiovascular events

*P < 0.05 compared with HS≦7% and NT-proBNP≦median group

†P < 0.05 compared with HS≦7% and NT-proBNP >median group

#P < 0.05 compared with HS>7% and NT-proBNP≦median group

^&^*P* < 0.05 compared with HS>7% and NT-proBNP >median group

The median of NT-proBNP cut at 261.80 pg/ml

**Table 4:** The risks for commencing dialysis and rapid eGFR decline according to plasma N-terminal pro-brain natriuretic peptide (NT-proBNP) and fluid status.

	Commencing dialysis				Rapid eGFR decline			
	Unadjusted Hazard ratio (95% Cl)	P-value	Adjusted Hazard ratio (95% Cl)	P-value	Unadjusted Odds ratio (95% Cl)	P-value	Adjusted Odds ratio (95% Cl)	P-value
HS >7%	2.35(1.65–3.34)	<0.001	1.53(1.02–2.28)	0.039	2.89(1.51–4.45)	0.001	1.87(0.95–3.70)	0.071
Log(NT-proBNP)	2.16(1.59–2.94)	<0.001	1.44(1.02–2.03)	0.038	1.37(0.88–2.15)	0.168	1.13(0.60–2.11)	0.705
HS≦7%, NT-proBNP≦median	Reference		Reference		Reference		Reference	
HS≦7%, NT-proBNP >median	1.94(1.13–3.31)	0.016	1.18(0.68–2.06)	0.555	2.26(0.99–5.16)	0.053	0.83(0.30–2.26)	0.710
HS>7%, NT-proBNP ≦median	1.50(0.89–2.53)	0.128	1.49(0.81–2.74)	0.201	0.75(0.33–1.72)	0.502	1.55(0.59–4.09)	0.378
HS>7%, NT-proBNP >median	3.32(2.14–5.16)	<0.001	1.71(1.03–2.85)	0.038	2.40(1.25–4.63)	0.009	1.90(0.79–4.52)	0.150

The median of NT-proBNP cut at 261.80 pg/ml

Abbreviations: CI, Confidence Interval; HS, hydration status; eGFR, estimated glomerular filtration rate

Adjusted model: age, sex, cardiovascular disease, diabetes mellitus, diuretics usage, angiotensin converting enzyme inhibitors/angiotensin II receptor blockers use, estimated glomerular filtration rate, and urine protein-creatinine ratio

**Fig 3:**
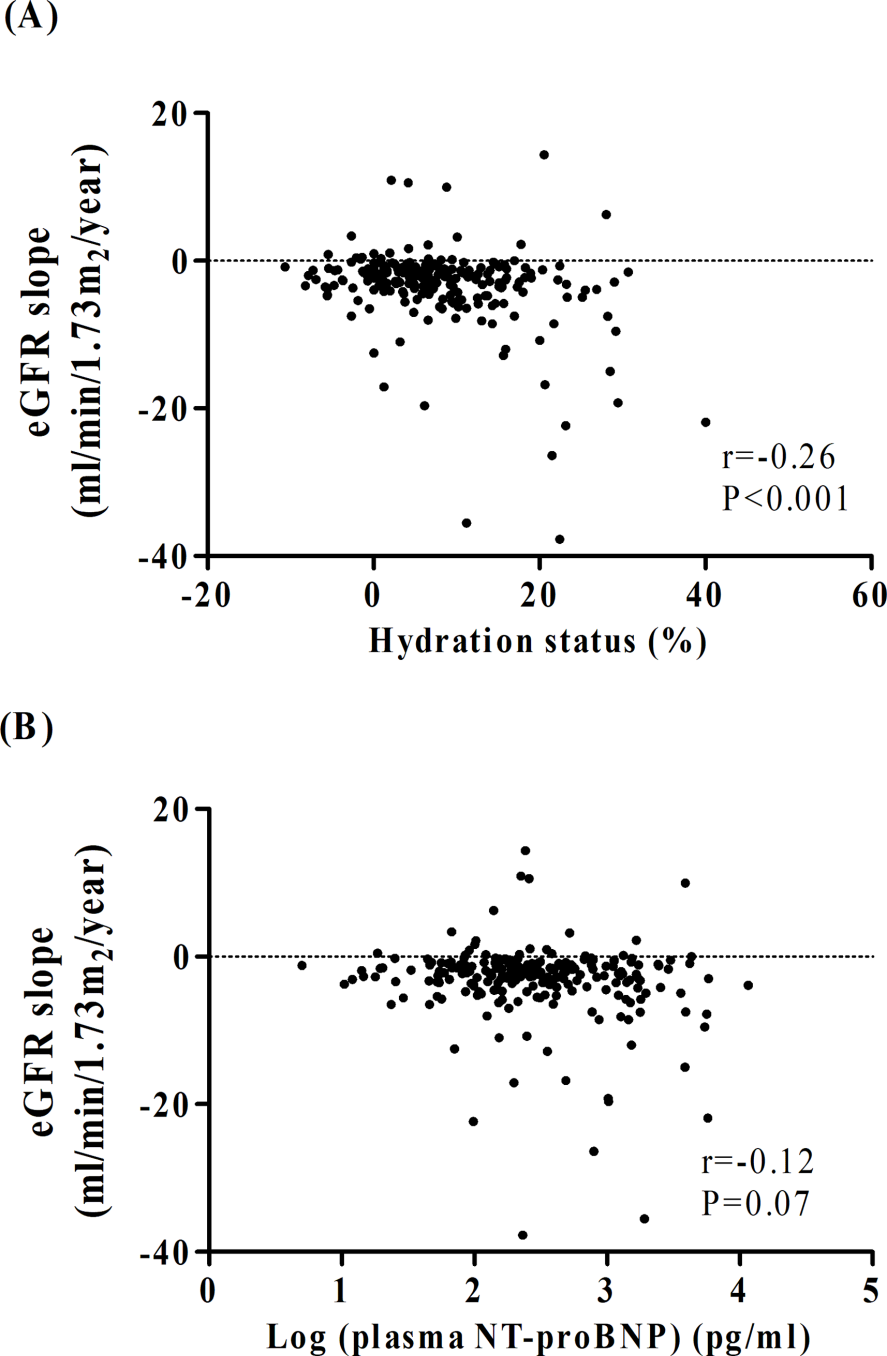
The association of eGFR decline with (A) fluid status and (B) plasma N-terminal pro-brain natriuretic peptide (NT-proBNP).

Eighty-eight (37.3%) patients had rapid eGFR decline during the follow-up period. Fig 3 reveal the negative association of eGFR decline with HS or NT-proBNP. Patients with both HS>7% and high circulating NT-proBNP level had the greatest renal function decline (eGFR slope: -3.1(-6.1,-1.4) mL/min/1.73 m^2^/year, Table 3) among the four groups. Both HS>7% and high circulating NT-proBNP were synergistically associated with rapid eGFR decline in unadjusted model (2.40, 95% CI: 1.25–4.63), but not in adjusted analysis (Table 4). There was no significant interaction between HS and circulating NT-proBNP level in rapid renal function decline (P-interaction = 0.527).

### Fluid status, NT-proBNP, MACEs and all-cause mortality

Of all patients, forty-eight patients (20.3%) reached MACEs or all-cause mortality (Table 3) before commencing dialysis. Thirty-one had MACEs, 23 died, and 6 had both MACEs and mortality during the follow-up period. The causes of mortality included three cardiovascular events, 11 sepsis, three malignancy, three refusing dialysis and the rest were a mixture of other causes. The causes of MACEs were events of nine acute myocardial infarction, nine acute hemorrhagic or ischemic stroke, 12 hospitalizations due to congestive heart failure, one sick sinus syndrome and one aortic dissection. There were no significant differences in the proportion of MACEs and all-cause mortality between the four groups.

Patients with both HS>7% and high circulating NT-proBNP level had higher risk for reaching MACEs or all-cause mortality than others (Fig 2B). Not only HS (HR: 1.08, 95%CI: 1.03–1.13) but also log-transformed NT-proBNP (HR: 2.51, 95%CI: 1.27–4.95) was significantly associated with composite outcomes either MACEs or all-cause mortality (Table 5). The consistent significance was also in cox-proportional and multivariate analysis for MACEs or all-cause mortality alone. The adjusted HR of composite outcomes either MACEs or all-cause mortality was 2.41 (95% CI: 1.01–5.77) for patients of both HS>7% and high circulating NT-proBNP level compared with those with HS≦7% and low circulating NT-proBNP level. However, there was no significant result in analysis for only MACEs or all-cause mortality. No significant interaction between HS and circulating NT-proBNP level in MACEs or all-cause mortality was found.

**Table 5:** The adjusted risks for major adverse cardiovascular events (MACEs) and all-cause mortality according to plasma N-terminal pro-brain natriuretic peptide (NT-proBNP) and fluid status.

	MACEs + all-cause mortality		MACEs		All-cause mortality	
	Hazard ratio (95% Cl)	P-value	Hazard ratio (95% Cl)	P-value	Hazard ratio (95% Cl)	P-value
HS, %	1.08(1.03–1.13)	0.002	1.07(1.02–1.13)	0.012	1.07(0.99–1.14)	0.098
Log(NT-proBNP)	2.51(1.27–4.95)	0.008	1.92(0.86–4.26)	0.110	3.27(1.13–9.52)	0.030
HS≦7%, NT-proBNP≦median	Reference		Reference		Reference	
HS≦7%, NT-proBNP >median	1.32(0.48–3.58)	0.591	1.06(0.31–3.63)	0.923	1.67(0.34–8.23)	0.527
HS>7%, NT-proBNP ≦median	1.64(0.55–4.83)	0.375	1.26(0.32–4.91)	0.738	0.91(0.16–5.06)	0.910
HS>7%, NT-proBNP >median	2.41(1.01–5.77)	0.048	2.07(0.94–1.41)	0.176	2.61(0.72–9.42)	0.102

The median of NT-proBNP cut at 261.80 pg/ml

Abbreviations: CI, Confidence Interval; eGFR, estimated glomerular filtration rate

Adjusted model: age, sex, cardiovascular disease, diabetes mellitus, diuretics usage, angiotensin converting enzyme inhibitors/angiotensin II receptor blockers usage, statin usage, estimated glomerular filtration rate, and urine protein-creatinine ratio, low-density lipoprotein

We also used urine PCR cut 1mg/mg in multivariable model of adverse outcomes for sensitivity analysis (S1 and S2 Tables).

## Discussion

This study evaluated the interaction between plasma NT-proBNP level and fluid status in adverse clinical outcomes in patients with CKD stages 4–5 over an observation period of 3.3 years. CKD patients with both fluid overload and high plasma NT-proBNP level had more increased risk for commencing dialysis and composite outcomes either MACEs or all-cause mortality than others with only fluid overload or high plasma NT-proBNP level. NT-proBNP and fluid overload had a synergistic activity of prediction of adverse clinical outcomes in late stage of CKD.

Fluid overload causes cardiomyocyte elongation and leads to LV remodeling and decompensation [23,24]. Then, BNP will be synthesized from cardiomyocyte in response to increased stress and stretch of LV wall [1,2]. Therefore, NT-proBNP level has been considered as an indicator of intravascular fluid status [25, 26]. NT-proBNP was also correlated with fluid volume imbalance between ICW and ECW [16]. Our results found patients with both high HS and high NT-proBNP had higher NT-proBNP level than those with only high NT-proBNP without high HS, meaning that fluid status has an impact on the increase in NT-proBNP. Accumulating evidences show renal function affects circulating NT-proBNP level [27]. Deterioration of renal function increased NT-proBNP/BNP ratio [27]. Besides, underlying cardiovascular disease also has an impact on NT-proBNP level. Thus, we add baseline renal function and cardiovascular disease in adjusted model for analysis, and the results reveal a close correlation between fluid overload and NT-proBNP. NT-proBNP is positively associated with fluid status independent of renal function and underlying cardiovascular disease in late stages of CKD.

NT-proBNP and fluid overload share many pathophysiologic mechanisms of cardiorenal syndrome, such as excess activation of renin-angiotensin-aldosterone system (RAAS), impaired immunologic system and inflammation and [20, 28–31]. Activation of RAAS is involved in the impairment of LV function resulting in elevation of NT-proBNP [28], and RAAS also modulates hemodynamic stability by regulating blood pressure and fluid status [30]. The pro-inflammatory cytokines interleukin-1beta and tumor necrosis factor-alpha leads to a selective increase of BNP gene expression and secretion [31], and NT-proBNP is associated with inflammatory markers, such as interleukin-6 and CRP [32,33]. Otherwise, inflammation has a close link with fluid status [20]. Fluid overload increases gut permeability, further destroying protective barriers and causing overgrowth of pathogenic species [34]. Therefore, the interaction between NT-proBNP and fluid overload might be correlated with poor clinical prognosis. We demonstrated a synergistic association of NT-proBNP and fluid overload with adverse clinical outcomes. Both NT-proBNP and fluid status could provide greater predictive activity of adverse clinical outcomes in CKD population.

Besides, baseline renal function and proteinuria are traditional predictors of adverse clinical outcomes in CKD patients. Fluid status and plasma NT-proBNP both increase progressively with the decline in renal function and the increase in proteinuria [13, 35]. Based on this close correlation, we adjusted baseline renal function and the severity of proteinuria in the analysis model, and fluid status and NT-proBNP could predict disease progression. Besides, after adjusting well-known risk factors of poor clinical outcomes, such as aging, sex, underlying cardiovascular disease and diabetes mellitus, CKD patients with both high plasma NT-proBNP and fluid overload still had increased risks for commencing dialysis, MACEs or all-cause mortality. NT-proBNP and fluid status could be utilized to monitor cardiac-renal axis in clinical CKD practice.

NT-proBNP has been considered as a tool of the diagnostic and prognostic activities in patients with heart failure or CKD on dialysis or not [36–39]. Furthermore, numerous studies report NT-proBNP can be utilized to monitor the efficiency of heart failure [40, 41]. A NT-proBNP-guided management strategy can reduce mortality in patients with heart failure [40]. Besides, BNP also has a clinical utility to evaluate the effect of Beta-blocker on LV diastolic function in patients on dialysis [42]. Because of the close correlation between NT-proBNP and fluid status, NT-proBNP may help clinical physicians to evaluate the current fluid status and the effect of diuretics usage on fluid overload in CKD patients. Fluid status may be controlled by a NT-proBNP-driven management strategy, further ameliorating cardiovascular burdens in patients on dialysis. Similarly, fluid status assessed by BCM has been reported to assist in regression of left ventricular mass index and arterial stiffness, as well as decrease in blood pressure in hemodialysis patients [43]. However, the effect of a NT-proBNP or BCM-guided management strategy on adverse clinical outcomes remains to be determined in CKD patients not on dialysis. Further study is necessary to identify the influence of NT-proBNP and BCM-driven intervention on cardiovascular consequence, all-cause mortality and poor renal progression.

NT-proBNP and fluid overload have been considered as a biomarker of MACEs and all-cause mortality [14, 44, 45]. The consistent results are also shown in the present study, but we did not find the significant synergistic association of NT-proBNP and fluid overload with MACEs or all-cause mortality. The relatively small number of cohort and events of this study is one of the reasons why the statistical power is diminished. Beyond NT-proBNP and fluid status, uncertain mechanisms might participate in increased risks for MACEs and all-cause mortality in CKD patients. Besides, this study also has some limitations. Fluid status, NT-proBNP, and diuretics usage were measured once at enrollment. The association of time-varying fluid status and NT-proBNP with clinical outcomes could not be estimated. Additionally, urine sodium and sodium intake were not examined or recorded in the study, whereas positive sodium balance might affect fluid status, vascular pressure and NT-proBNP level [44]. The effect of sodium retention on fluid status and clinical outcomes might be underestimated.

## Conclusions

Our study demonstrates that fluid overload and NT-proBNP are synergistically associated with commencing dialysis in patients with CKD stages 4–5. Patients with both fluid overload and high circulating NT-proBNP level had greater risks for MACEs or all-cause mortality than those with either fluid overload or high circulating NT-proBNP level. The interaction between NT-proBNP and fluid status in adverse outcomes should be considered in clinical care of late stages of CKD, and could be utilized in prediction of clinical outcomes more precisely.

## Supporting information

S1 Table Sensitivity analysis of the risks for commencing dialysis and rapid eGFR decline according to plamsa N-terminal pro-brain natriuretic peptide (NT-proBNP) and fluid status. (DOCX)

S2 Table Sensitivity analysis of the adjusted risks for major adverse cardiovascular events (MACEs) and all-cause mortality according to plasma N-terminal pro-brain natriuretic peptide (NT-proBNP) and fluid status. (DOCX)
