# Supplementary material for: The interaction between N-terminal pro-brain natriuretic peptide and fluid status in adverse clinical outcomes of late stages of chronic kidney disease
Source: PLoS One. 2018 Aug 22;13(8):e0202733. doi: 10.1371/journal.pone.0202733 (PMC6105012; doi:10.1371/journal.pone.0202733)
Supplement: S1 Table — (DOCX) [file pone.0202733.s001.docx]

S1 Table. Sensitivity analysis of the risks for commencing dialysis and rapid eGFR decline according to plamsa N-terminal pro-brain natriuretic peptide (NT-proBNP) and fluid status

|  | Commencing dialysis | |  |  | Rapid eGFR decline | |  |  |
| --- | --- | --- | --- | --- | --- | --- | --- | --- |
|  | Unadjusted  Hazard ratio  (95% Cl) | P-value | Adjusted  Hazard ratio  (95% Cl) | P-value | Unadjusted  Odds ratio  (95% Cl) | P-value | Adjusted  Odds ratio  (95% Cl) | P-value |
| HS, % | 1.06(1.04-1.08) | 0.001 | 1.04(1.01-1.06) | 0.002 | 1.06(1.03-1.10) | <0.001 | 1.07(1.03-1.12) | 0.001 |
| Log-formed NT-proBNP, pg/ml | 2.16(1.59-2.94) | <0.001 | 1.62(1.13-2.32) | 0.009 | 1.37(0.88-2.15) | 0.168 | 1.61(0.89-2.90) | 0.116 |
| HS≦7%, NT-proBNP≦median | Reference |  | Reference |  | Reference |  | Reference |  |
| HS>7%, NT-proBNP ≦median | 1.50(0.89-2.53) | 0.128 | 1.23(0.70-2.15) | 0.467 | 0.75(0.33-1.72) | 0.502 | 0.79(0.29-2.14) | 0.636 |
| HS≦7%, NT-proBNP >median | 1.94(1.13-3.31) | 0.016 | 1.93(1.07-3.49) | 0.029 | 2.26(0.99-5.16) | 0.053 | 1.99(0.77-5.14) | 0.158 |
| HS>7%, NT-proBNP >median | 3.32(2.14-5.16) | <0.001 | 2.11(1.30-3.42) | 0.003 | 2.40(1.25-4.63) | 0.009 | 2.53(1.09-5.90) | 0.031 |

The median of NT-proBNP cut at 261.80 pg/ml

Abbreviations: CI, Confidence Interval; HS, hydration status; eGFR, estimated glomerular filtration rate

Adjusted model: age, sex, cardiovascular disease, diabetes mellitus, diuretics usage, angiotensin converting enzyme inhibitors/angiotensin II receptor blockers use, estimated glomerular filtration rate, and urine protein-creatinine ratio cut at 1g/g
