# Supplementary material for: The interaction between N-terminal pro-brain natriuretic peptide and fluid status in adverse clinical outcomes of late stages of chronic kidney disease
Source: PLoS One. 2018 Aug 22;13(8):e0202733. doi: 10.1371/journal.pone.0202733 (PMC6105012; doi:10.1371/journal.pone.0202733)
Supplement: S2 Table — (DOCX) [file pone.0202733.s002.docx]

S2 Table. Sensitivity analysis of the adjusted risks for major adverse cardiovascular events (MACEs) and all-cause mortality according to plasma N-terminal pro-brain natriuretic peptide (NT-proBNP) and fluid status

|  | MACEs + all-cause mortality | | MACEs |  | All-cause mortality | |
| --- | --- | --- | --- | --- | --- | --- |
|  | Hazard ratio  (95% Cl) | P-value | Hazard ratio  (95% Cl) | P-value | Hazard ratio  (95% Cl) | P-value |
| HS, % | 1.09(1.04-1.13) | <0.001 | 1.08(1.02-1.14) | 0.005 | 1.08(1.00-1.15) | 0.046 |
| Log-formed NT-proBNP | 2.78(1.40-5.48) | 0.003 | 2.08(0.93-4.65) | 0.073 | 3.82(1.32-11.02) | 0.013 |
| HS≦7%, NT-proBNP≦median | Reference |  | Reference |  | Reference |  |
| HS>7%, NT-proBNP ≦median | 1.45(0.53-3.94) | 0.467 | 1.08(0.31-3.69) | 0.907 | 1.69(0.34-8.44) | 0.524 |
| HS≦7%, NT-proBNP >median | 1.74(0.59-5.11) | 0.317 | 1.35(0.35-5.25) | 0.664 | 1.07(0.19-5.94) | 0.941 |
| HS>7%, NT-proBNP >median | 2.71(1.11-6.58) | 0.028 | 2.35(0.81-6.84) | 0.116 | 2.71(0.73-10.15) | 0.138 |

The median of NT-proBNP cut at 261.80 pg/ml

Abbreviations: CI, Confidence Interval; eGFR, estimated glomerular filtration rate

Adjusted model: age, sex, cardiovascular disease, diabetes mellitus, diuretics usage, angiotensin converting enzyme inhibitors/angiotensin II receptor blockers usage, estimated glomerular filtration rate, urine protein-creatinine ratio cut at 1g/g, and low-density lipoprotein
